# Comprehensive analysis of genomic complexity in the 5’ end coding region of the *DMD* gene in patients of exons 1–2 duplications based on long-read sequencing

**DOI:** 10.1186/s12864-024-10224-2

**Published:** 2024-03-19

**Authors:** Jiandong Shen, Taoli Ding, Xueping Sun, Ji Yang, Yue Zhang, Jing Wang, Mengdi Ge, Heng Xu, Jiazi Xie, Fei Wang, Feiyang Diao

**Affiliations:** 1 State Key Laboratory of Reproductive Medicine and Offspring Health, Clinical Center of Reproductive Medicine, First Affiliated Hospital, Nanjing Medical University, Jiangsu Province, Nanjing, 210029 China; 2 Yikon Genomics Company, Ltd, Jiangsu Province, Suzhou, 215000 China

**Keywords:** *DMD*, Long-read sequencing (LRS), Exonic duplications, Dp427c/Dp427m

## Abstract

**Background:**

Dystrophinopathies are the most common X-linked inherited muscle diseases, and the disease-causing gene is *DMD*. Exonic duplications are a common type of pathogenic variants in the *DMD* gene, however, 5’ end exonic duplications containing exon 1 are less common. When assessing the pathogenicity of exonic duplications in the *DMD* gene, consideration must be given to their impact on the reading frame. Traditional molecular methods, such as multiplex ligation-dependent probe amplification (MLPA) and next-generation sequencing (NGS), are commonly used in clinics. However, they cannot discriminate the precise physical locations of breakpoints and structural features of genomic rearrangement. Long-read sequencing (LRS) can effectively overcome this limitation.

**Results:**

We used LRS technology to perform whole genome sequencing on three families and analyze the structural variations of the *DMD* gene, which involves the duplications of exon 1 and/or exon 2. Two distinct variant types encompassing exon 1 in the *DMD* Dp427m isoform and/or Dp427c isoform are identified, which have been infrequently reported previously. In pedigree 1, the male individuals harboring duplication variant of consecutive exons 1–2 in the *DMD* canonical transcript (Dp427m) and exon 1 in the Dp427c transcript are normal, indicating the variant is likely benign. In pedigree 3, the patient carries complex SVs involving exon 1 of the *DMD* Dp427c transcript showing an obvious phenotype. The locations of the breakpoints and the characteristics of structural variants (SVs) are identified by LRS, enabling the classification of the variants' pathogenicity.

**Conclusions:**

Our research sheds light on the complexity of *DMD* variants encompassing Dp427c/Dp427m promoter regions and emphasizes the importance of cautious interpretation when assessing the pathogenicity of *DMD* 5' end exonic duplications, particularly in carrier screening scenarios without an affected proband.

**Supplementary Information:**

The online version contains supplementary material available at 10.1186/s12864-024-10224-2.

## Background

Dystrophinopathies are the most common X-linked inherited muscle diseases, and the manifestations range from mild phenotypes of asymptomatic increase in serum concentration of creatine phosphokinase (CK) to severe phenotypes that include Duchenne muscular dystrophy (DMD, MIM 310200), Becker muscular dystrophy (BMD, MIM 300376), and *DMD*-associated dilated cardiomyopathy (DCM, MIM 302045) [1]. DMD usually presents in early childhood, and affected children are often wheelchair dependent by age 12 years, and few survive beyond the third decade, with respiratory complications and progressive cardiomyopathy being common causes of death. BMD is characterized by later-onset and relatively slow progress, and heart failure from DCM is a common cause of death in BMD [2, 3]. The exact prevalence data of dystrophinopathies are not available. DMD is more common than BMD, and it is reported that the incidence of DMD is 1:4,700 live male births in Canada [4] and 1:3,917 live male births in southeast Norway [5].

The molecular basis of DMD/BMD and DCM is pathogenic variation in the *DMD* gene (MIM 300377), the largest gene in humans, spanning 2.2 Mb genome sequence at Xp21, consisting of 79 exons. The *DMD* gene contains at least seven independent, tissue-specific promoters and two polyA-addition sites [6]. Among these, three full-length isoforms share the same number of exons but are derived from three independent promoters (exon 1) in the brain (Dp427c), muscle (Dp427m), and Purkinje cerebellar neurons (Dp427p) [2]. While many variants have been documented within this gene, a majority of them affect the expression of the muscle isoform (Dp427m) [2]. About 65% of *DMD* gene pathogenic variants are exonic deletions, ~ 10% are exonic duplications, and about 25% are small variants, including point mutations, small insertions/deletions (indels), and others [3]*.*

Numerous molecular genetic methods are available for mutation screening of the *DMD* gene. Multiple polymerase chain reaction (M-PCR), targeting mutation hotspots, can detect approximately 98% of exonic deletions [7]. Multiplex ligation-dependent probe amplification (MLPA) [8] is more widely used in clinical labs for *DMD* mutation screening because it can simultaneously detect exonic deletions and duplications. The next-generation sequencing (NGS) technology enables rapid and comprehensive screening of single nucleotide variations (SNVs) and small indels among 79 exons in the *DMD* gene. Genetic diagnosis could be confirmed in around 98% of DMD/BMD patients by MLPA combined with NGS technology [9]. However, the traditional method of mutation screening for *DMD* cannot identify the complex structural variants (SVs) of the *DMD* gene, such as discerning whether *DMD* exonic duplications occur extragenically or intragenically and whether in tandem or not. This information holds significance for determining the pathogenicity of duplications [10]. Recently, long-read sequencing (LRS) methods have emerged, which can generate genome assemblies of unprecedented quality. Leveraging the advantages of longer reads, LRS has been successfully employed in the genetic testing of monogenic diseases with structural complexity, including thalassemia [11] and congenital adrenal hyperplasia [12].

In this study, we selected three unique families with duplication variants affecting exon 1 and/or exon 2 in the *DMD* gene to explore the structural characteristics of exonic duplications through LRS. Our investigation aimed to shed light on the pathogenicity of these variants and provide further insights into their implications.

## Results

### MLPA results

**Fig. 1:**
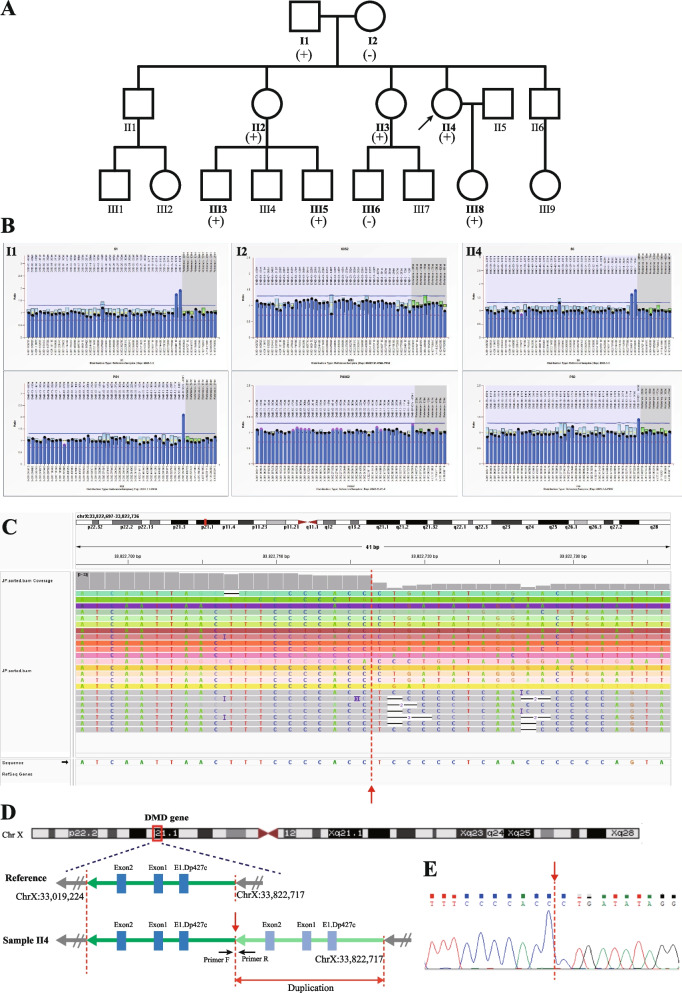
Genetic analysis of the *DMD* gene in Pedigree 1. **A** and **B** show the family pedigree and *DMD* gene analysis results detected by MLPA, male members I1, III3, and III5 had hemizygous duplication variants of consecutive *DMD* exons 1–2 in Dp427m and exon 1 in Dp427c, and female members II2, II3, II4 and III8 were heterozygous. **C** shows a critical breakpoint from a screenshot of the integrative genomics viewer (IGV) based on LRS data analysis. **D** Schematic diagram shows the location of the breakpoint and architectural features of the duplication variant. E is the result of Sanger sequencing verification for the critical breakpoint. The red dashed line indicates the breakpoints and the red single arrow indicates the same critical breakpoint

In pedigree 1, the index patient (II4) was identified with a duplication of exon 1–2 in the *DMD* gene during routine expanded carrier screening (ECS). Subsequently, MLPA was used to confirm that the duplication occurred in exons 1–2 in the Dp427m isoform and exon 1 in the Dp427c isoform of the *DMD* gene. Further investigation of the family demonstrated that the other three females (II2, II3, and III8) were heterozygous duplication carriers. Unexpectedly, three male members (I1, III3, III5) harbored the same hemizygous duplication variants, without clinical manifestations of DMD/BMD and abnormal biochemical indicators (I1, III3). The lack of genotype–phenotype cosegregation suggested that the duplication variant affecting exons 1–2 in the Dp427m isoform and exon 1 in the Dp427c isoform of the *DMD* gene was likely benign. (See Fig. 1B and Supplementary Fig. 1).

**Fig. 2:**
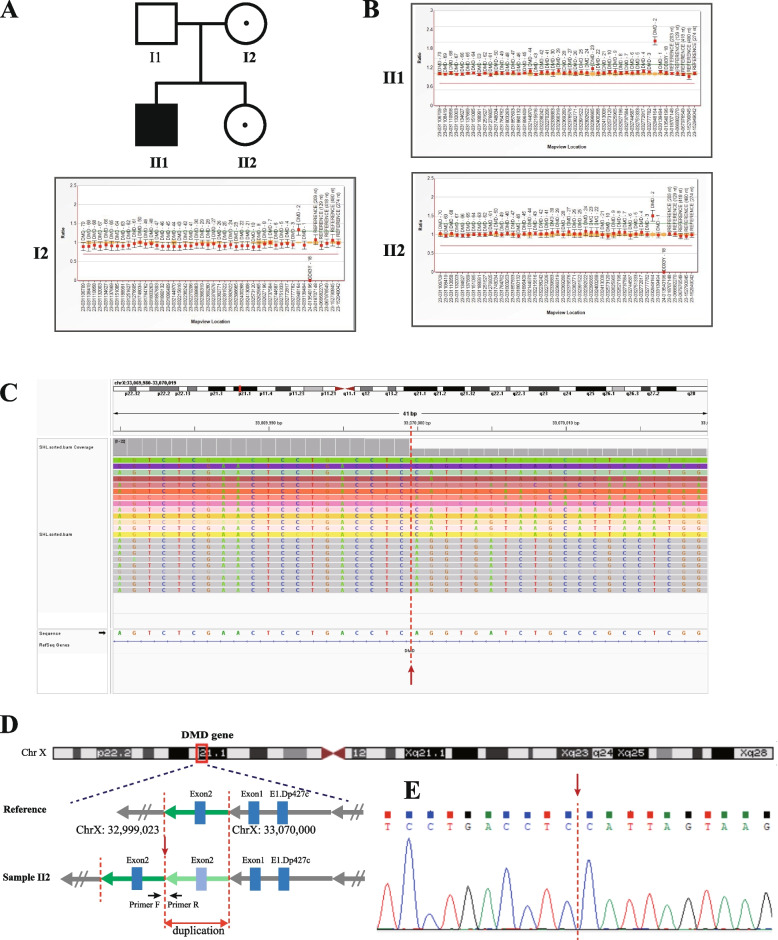
Genetic analysis of the *DMD* gene in Pedigree 2. **A** and **B** show the family pedigree and *DMD* gene analysis results detected by MLPA, affected boy (II1) had a hemizygous duplication variant of exon 2 in the *DMD* gene, and his mother and sisiter were heterozygous. **C** shows a critical breakpoint from a screenshot of the integrative genomics viewer (IGV) based on LRS data analysis. **D** Schematic diagram shows the locations of breakpoints and architectural features of the duplication variants. E is the result of Sanger sequencing verification for the critical breakpoint. The red dashed line indicates the breakpoints and the red single arrow indicates the same critical breakpoint

In pedigree 2, the affected boy (II1) presented with a duplication variant involving exon 2 in the *DMD* gene, displaying the characteristic clinical phenotype of DMD. His sister (II2, index patient) carried the heterozygous duplication of exon 2, with mild phenotype of abnormal biochemical indicators. Their mother (I2) was identified as a carrier of the heterozygous duplication variant of exon 2 in the *DMD* gene, without abnormal phenotype. (See Fig. 2B).

**Fig. 3:**
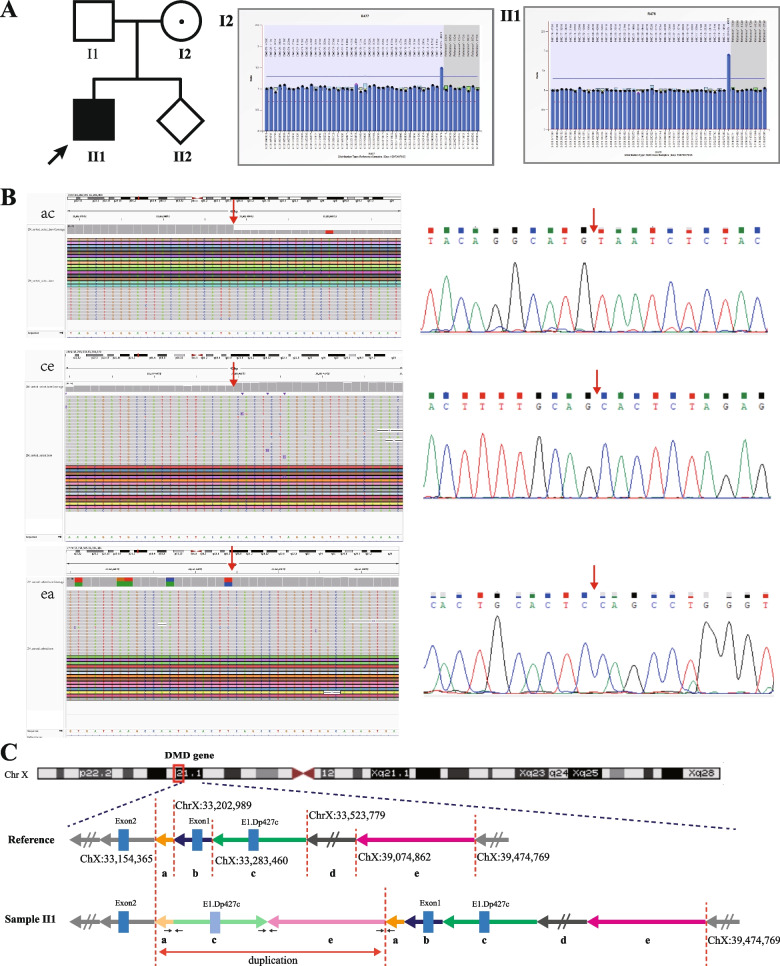
Genetic analysis of the *DMD* gene in Pedigree 3. **A** shows the family pedigree and *DMD* gene analysis results detected by MLPA. The proband (II1) had hemizygous duplication variants of exon 1 of the Dp427c transcript in the *DMD* gene, and his mother was heterozygous. **B** shows critical breakpoints from screenshots of the integrative genomics viewer (IGV) based on LRS data analysis, and corresponding verification results by Sanger sequencing. From top to bottom are the joints of fragments a and c, fragments c and e, fragments e and a (indicated in **C**). The red single arrows indicate the breakpoints. **C** Schematic diagram shows the locations of breakpoints and architectural features of the duplication variant. The red dashed lines indicate the breakpoints involved in recombination, and the coordinates of the breakpoints in the genome are shown next to them

In pedigree 3, the proband exhibited a duplication variant involving exon 1 in Dp427c, displaying typical clinical phenotypes of DMD. His pregnant mother was identified as a carrier of the heterozygous variant. (See Fig. 3A).

### Breakpoints and architectural features identification by LRS and validation

Whole genome LRS was performed to identify the breakpoints, and the sequencing data parameters were described in Supplementary Table 2. In the index patient (II4) in pedigree 1, LRS revealed that the duplication variant was located at chrX:33,019,224–33,822,717. This duplication encompassed a contiguous segment of approximately 803.5 kb, spanning consecutive *DMD* exons 1–2 within the Dp427m transcript and exon 1 within the Dp427c transcript. The duplication occurred in a tandem arrangement. The critical breakpoint was confirmed by Sanger sequencing. (See Fig. 1C\D\E).

In pedigree 2, the fresh lymphocyte sample was unavailable from the affected boy (II1), but a fresh sample was obtained from II2. LRS on II2 indicated the duplication variant was ~ 71.0 kb, located at chrX:32,999,023–33,070,000. This duplication involved a single exon 2 of the *DMD* gene and was arranged in tandem, potentially disrupting the reading frame of the *DMD* gene. We conducted Sanger sequencing to validate the critical breakpoint. (See Fig. 2C\D\E).

In pedigree 3, the proband (II1) exhibited a complex duplication spanning approximately 688.9 kb within the DMD gene, as identified by LRS. This duplication involved an inverted single exon 1 of the Dp427c transcript. The initial tandem duplication segment was potentially substantial (~ 6.3 Mb), ranging from chrX:33,154,365 to chrX:39,474,769. Additionally, two internal deletions were observed either following or occurring simultaneously with the tandem duplication. These deletions encompassed fragments b (chrX:33,202,989–33,283,460) and d (chrX:33,523,779–39,074,862), respectively. Furthermore, an inversion (fragment c, chrX:33,283,460–33,523,779) was also detected. These complex genomic rearrangements result in an out-of-frame variant. Three critical breakpoints were verified by Sanger sequencing. (See Fig. 3 B\C).

No pathogenic SNVs and indels were identified in any of the subjects based on nanopore sequencing data. The size of duplication variants revealed by LRS was consistent with those determined by CNV-Seq using NGS with a resolution of 100 kb. (See Supplementary Fig. 2).

### Sequence characteristics of breakpoints

In the three pedigrees examined in this study, repeat sequences were observed surrounding most of the breakpoints. These include various types of repeat elements, such as short interspersed nuclear elements (SINE), long interspersed nuclear elements (LINE), long terminal repeat elements (LTR), and low complexity repeats. Detailed information can be found in Supplementary Fig. 3.

## Discussion

Exonic duplications are a frequent type of pathogenic variant in the *DMD* gene [3, 7, 13], and duplication of exon 2 is the most prevalent duplication variant among DMD patients [13]. While MLPA and NGS methods are commonly employed in clinical settings to detect exonic duplication variants in *DMD*, they often fail to discern the precise physical locations of breakpoints and structural characteristics of genomic rearrangements. However, LRS overcomes the limitations associated with assembly problems encountered when dealing with long and complex sequences. Kubota et al. [14] reported a DMD patient with complex genomic rearrangements involving exon 2 duplication through LRS, and simultaneously detected the normal intact *DMD* gene sequence, suggesting a mosaic nature in the patient. For individuals clinically diagnosed with DMD or BMD, long-read whole-genome sequencing presents a valuable approach for identifying potential structural variants within the *DMD* gene when conventional methods are unable to confirm the genetic diagnosis. Xie Z et al. [15] utilized this strategy to identify a DMD patient harboring a variant characterized by a large-scale inversion/deletion-insertion rearrangement in the *DMD* gene.

The relationship between genotype and phenotype in DMD/BMD patients is intricate. There is no apparent correlation between the size, region, domain, or mutations affecting splicing and the resulting severity. Instead, the primary determinant lies in maintaining the open reading frame, which enables the translation of a functional dystrophin protein [2]. When assessing the pathogenicity of exonic duplication variants within the *DMD* gene, it is essential to consider whether these variants disrupt the reading frame [13]. Tandem duplications of exons have the potential to disrupt the intact reading frame of the *DMD* gene, leading to disease manifestation. As in pedigree 2 in the present study, the duplicated exon 2 region was tandem and inserted into intron 1, which destroyed the integrated gene structure and potentially generated an aberrant transcript.

It has been reported recently, that cases with exonic duplications of *DMD* gene were accidentally found in the screening project, and eventually were proved to be benign variants. Bai Y et al*.* [10] reported that in the carrier screening program, a normal male was found to carry *DMD* exons 56–61 duplications, without DMD/BMD-related phenotype. The exonic duplications were confirmed to be external duplications of the *DMD* gene by LRS and did not affect the normal *DMD* gene structure. Similarly, He W et al*.* [16] reported that in the invasive prenatal diagnosis process, CNVs involving *DMD* gene fragments were found in chromosomal microarray analysis (CMA) examination, and the fetus was suggested to have duplications of exons 51–53 and exons 64–79 in the *DMD* gene, confirmed by MLPA. However, in the process of family validation, it was found that an asymptomatic male in the family carried the variant, and complex rearrangement duplications involving exons 51–53 and exons 64–79 of the *DMD* gene were revealed by LRS. Although the specific location of the variant was not clear, the individual exhibited an intact *DMD* gene structure, resulting in no abnormal phenotype in the carrier male.

In pedigree 1, the index patient (II4) was identified as carrying suspected pathogenic exonic duplications in the *DMD* gene by ECS. The duplicated segments contained exons 1–2 in the *DMD* canonical transcript (Dp427m) and exon 1 in the Dp427c transcript, which was confirmed by MLPA. Previously, exons 1–2 duplication variants in *DMD* detected by NGS had been reported [17]. Supplementary data provided by the authors revealed one case of a male patient with suspected DMD who harbored an exons 1–2 duplication variant (g. (33021155_33021205)_(33229754_33229804) dup), excluding exon 1 of the Dp427c transcript, with a size of approximately 208.5 kb. Another case was identified during carrier screening of females with a family history of DMD, where the female individual carried canonical exons 1–2 and exon 1 of Dp427c (g.(33020929_33020979)_(33439454_33439504) dup). However, no relevant family investigation information and breakpoint analysis in the *DMD* gene were provided in that research paper, making it unclear whether the variants were pathogenic and affected the normal reading frame [17].

In pedigree 1 of our study, three healthy adult male members (I1, III3, III5) carried the duplication variant, indicating its potentially benign nature. To further investigate, we tested lymphocyte samples from the index patient (II4) through LRS. Our findings revealed that although the duplication segment was tandem, it was inserted upstream of the Dp427c transcript of the *DMD* gene without disturbing the complete functional structure of the two transcripts (Dp427m and Dp427c) of the *DMD* gene. This suggests that the 5' end duplication in the *DMD* gene is likely benign, as it does not affect the complete functional structure of the *DMD* gene, despite being a tandem duplication.

In our local patient database, an intriguing observation was made in pedigree 3. We identified a typical male DMD patient who exhibited a duplication involving a single exon 1 in the *DMD* Dp427c transcript, as confirmed by MLPA. Considering the insight gained from pedigree 1, where the tandem duplication of exon 1 in Dp427c was likely not the actual disease-causing variant, further investigations were conducted. Consequently, we conducted LRS on the proband from pedigree 3. The LRS analysis revealed that the duplication fragment consisted of an inverted exon 1 from Dp427c and its insertion into intron 1 of the Dp427m transcript. This finding suggests that the duplication may indeed have an impact on the reading frame of the normal *DMD* gene. Unfortunately, obtaining muscle tissue from the patient for additional RNA analysis proved to be unfeasible.

Five potential mechanisms for genomic rearrangements were given: (i) homologous recombination, including non-allelic homologous recombination (NAHR), gene conversion, single strand annealing (SSA) and break-induced replication (BIR); (ii) non-homologous end joining (NHEJ); (iii) microhomology-mediated replication-dependent recombination (MMRDR); (iv) long interspersed element-1 (LINE-1)-mediated retrotransposition; and (v) telomere healing [18]. The instability of the *DMD* gene structure can be mediated by various factors present around the breakpoints, such as long motifs, nonconsensus microhomologies, low‐copy repeats, palindromic sequences, and microindels [19]. Consistent with a previous study [10], recurrent repeat sequences, such as SINE, LINE, and LTR, were observed around most of the breakpoints in the cases analyzed. These findings provide further support for the mechanistic hypothesis of MMRDR proposed in previous research.

## Conclusions

In this study, We used LRS technology to perform whole genome sequencing on three families and analyze the structural variations of the *DMD* gene. Firstly, we report for the first time that the duplication variant of consecutive exons 1–2 in the *DMD* canonical transcript (Dp427m) and exon 1 in the Dp427c transcript is likely benign. Additionally, we report for the first time a likely pathogenic duplication variant characterized by the inverted duplication of exon 1 from the Dp427c transcript within the deep intron 1 of the Dp427m transcript in the *DMD* gene. These novel insights enhance our understanding of the pathogenic variant spectrum associated with the *DMD* gene. Furthermore, our findings underscore the caution when interpreting the pathogenicity of 5' end exonic duplications in the *DMD* gene, particularly in carrier screening scenarios without a proband.

## Methods

### Subjects

Three pedigrees were collected in this study.

*Pedigree 1*, Index patient (II 4), a 37-year-old female, planned to undergo preimplantation genetic testing for structural rearrangement (PGT-SR) because of carrying a Robertsonian translocation chromosome. Before PGT-SR, the couple voluntarily selected expanded carrier screening (ECS) for recessive genetic diseases using a previously reported method [20], which suggested the female patient carried a duplication variant of exons 1–2 in the canonical isoform of the *DMD* gene. To analyze the pathogenicity of the variant and evaluate the reproductive risk, the family investigation was performed, and no members with DMD/BMD-related phenotypes were found. Relevant family members provided blood samples for further genetic testing. Among them, II4, I1, and III3 underwent routine biochemical tests, suggesting normal CK levels. (See Fig. 1A).

*Pedigree 2*, Index patient (II 2), a 9-year-old girl, physical examination revealed elevated CK (2192 U/L), slightly elevated aspartate aminotransferase (AST) (53.6U/L), and slightly elevated lactate dehydrogenase (LDH) (447U/L), but no abnormality was found in electromyography. She was referred to our clinic for a genetic diagnosis. Family history investigation suggested his brother was paralyzed when he was 12 years old and was clinically diagnosed with DMD by muscle biopsy without genetic testing. (See Fig. 2A).

*Pedigree 3*, Index patient (II1), at the age of 7 years, was clinically diagnosed with DMD because of progressively worsening weakness in both lower limbs and difficulty climbing stairs, and running. His serum CK, alanine aminotransferase (ALT), AST, and LDH levels were 9569 U/L, 284 U/L, 200 U/L, and 1282 U/L, respectively. Physical examination revealed positive Gower's sign and hypertrophy of the bilateral calf gastrocnemius muscles. At the age of 10 years, his mother took him to our clinic for a genetic diagnosis because his mother had a natural pregnancy and asked for a prenatal diagnosis. (See Fig. 3A).

Written informed consent was obtained from all participants and/or their legal guardian(s), and peripheral blood was taken. This study was approved by the Institutional Review Board of the First Affiliated Hospital of Nanjing Medical University (No. 2023-SR-454).

### DNA extraction

High molecular weight genomic DNA was extracted from peripheral blood samples using Blood & Cell Culture DNA Midi Kit (Qiagen, Germany), according to the standard operating procedure provided by the manufacturer. The degradation and contamination of the extracted DNA were assessed through 1% agarose gels. DNA purity was then detected using a NanoDrop™ One UV‒Vis spectrophotometer (Thermo Fisher Scientific, USA), of which the OD 260/280 ranged from 1.8 to 2.0 and the OD 260/230 was between 2.0 and 2.2. Finally, the DNA concentration was further measured by Qubit® 3.0 Fluorometer (Invitrogen, USA).

### Multiplex ligation-dependent probe amplification analysis

To investigate the exonic deletions and duplications in the *DMD* gene, multiplex ligation-dependent probe amplification (MLPA) was performed with SALSA MLPA Kit P034/P035 DMD/Becker (MRC-Holland, Amsterdam, Netherlands) according to the manufacturer’s protocol. Amplification products were analyzed by capillary electrophoresis on an ABI Prism 3100 genetic analyzer (Applied Biosystems, USA). The original data were analyzed by Coffalyser.net software (MRC-Holland,Amsterdam, Netherlands) according to the instructions.

### Library preparation and nanopore sequencing

A total amount of 2 µg DNA per sample was used as input material for the ONT library preparations. After the qualification, the size selection of long DNA fragments was performed using the BluePippin system (Sage Science, USA). Next, the ends of DNA fragments were repaired, and a ligation reaction was conducted with the NEBNext Ultra II End Repair/dA-tailing Kit (New England Biolabs, UK). The adapter in the LSK109 kit (Oxford Nanpore Technologies, UK) was used for further ligation reaction, and Qubit® 3.0 Fluorometer (Invitrogen, USA) was used to quantify the size of the library fragments. Sequencing was then performed on a PromethION sequencer (Oxford Nanopore Technologies, UK).

### Mapping, detection of variants

Raw data in fastq format were obtained by capturing the electrical signal generated by PromethION. Guppy basecalling software (v5.0.16) was employed during this process. To maintain analysis accuracy and data integrity, NanoFilt (v2.8.0, https://github.com/wdecoster/nanofilt) was applied to eliminate low-quality reads (Qphred <  = 7) and short reads (< 1000 bp) from the raw data. Additionally, a total of 50 bp bases from both the head and tail ends of the reads were trimmed. Minimap2 (https://github.com/lh3/minimap2) was employed to align the reads to the reference genomes hg19 (GRCh37) accurately. Subsequently, samtools (v1.2, https://github.com/samtools/samtools) was used to convert the resulting SAM file to the BAM format for further processing and analysis. Sniffles2 (https://github.com/fritzsedlazeck/Sniffles) was utilized to process the BAM files to detect structural variations (SVs) in the genomic data. To refine the results, screening based on high-quality variant reads was conducted, and the karyotype diagnosis report was examined. By combining these analyses, preliminary SV results with improved accuracy and reliability were obtained. To examine single nucleotide variants (SNVs) and indels from samples, PEPPER-Margin-DeepVariant (r0.8-gpu, https://github.com/kishwarshafin/pepper) was employed by providing the BAM file as input.

### Analysis of sequence characteristics near breakpoints

A manual analysis of the flanking sequences at each breakpoint was performed to investigate the presence of repetitive elements, and 100 or 200-base pair reads both upstream and downstream of each breakpoint were extracted. The "Repeat Masker" program from the UCSC Genome Browser was employed to conduct a comprehensive search for repetitive elements within these extracted reads.

### NGS sequencing and copy number variants (CNVs) analysis

Approximately 50 ng of genomic DNA (gDNA) underwent fragmentation using the DNA Fragment kit (KT100804248, Yikon, China), followed by library preparation using the DNA library prepare kit (XK038, Yikon, China). The quality of the resulting library was assessed using the Agilent 2100 Bioanalyzer (Agilent, USA). Subsequently, DNA libraries were subjected to sequencing on the Nextseq500 system (Illumina, USA). Copy number quantification across the genome was performed using NGS reads, following established protocols.

### PCR and Sanger sequencing validation

The breakpoints of the *DMD* gene identified by nanopore sequencing were confirmed by PCR and Sanger sequencing. PCR primers were designed using MFEprimer-3.1 (https://mfeprimer3.igenetech.com/), and primer sequences were listed in Supplementary Table 1. Template gDNA was amplified using 25 ul 2 × GoldStar Best MasterMix (CW0655M, Cwbio, China), 2 ul forward primer, and 2 ul reverse primer to obtain around 1 kb PCR products. PCR products were confirmed on agarose gels and sequenced on an ABI Prism 3730xl Genetic Analyser (Applied Biosystems) and analyzed with Chromas software (Technelysium, Australia).

### Supplementary Information

**Supplementary Material 1. **

**Supplementary Material 2. **

**Supplementary Material 3. **

**Supplementary Material 4. **

**Supplementary Material 5. **

## Data Availability

The data that support the findings of this study have been deposited into the CNGB Sequence Archive (CNSA) of China National GeneBank DataBase (CNGBdb) with accession number CNP0004854. (https://db.cngb.org/search/project/CNP0004854/).
