# Supplementary material for: Comprehensive analysis of genomic complexity in the 5’ end coding region of the DMD gene in patients of exons 1–2 duplications based on long-read sequencing
Source: BMC Genomics. 2024 Mar 19;25:292. doi: 10.1186/s12864-024-10224-2 (PMC10949565; doi:10.1186/s12864-024-10224-2)

**Supplementary Figure 1**: MLPA charts of all the available members in pedigree 1

**I1：**


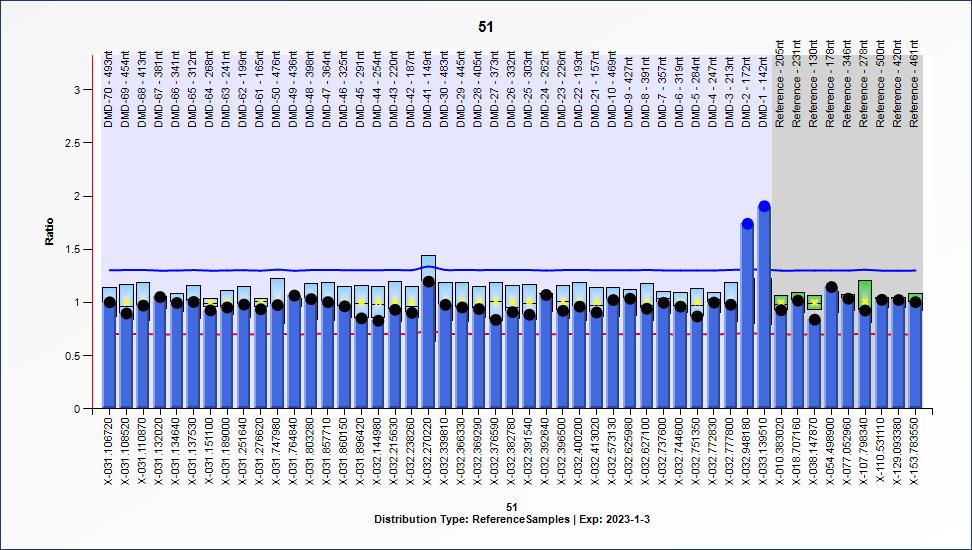

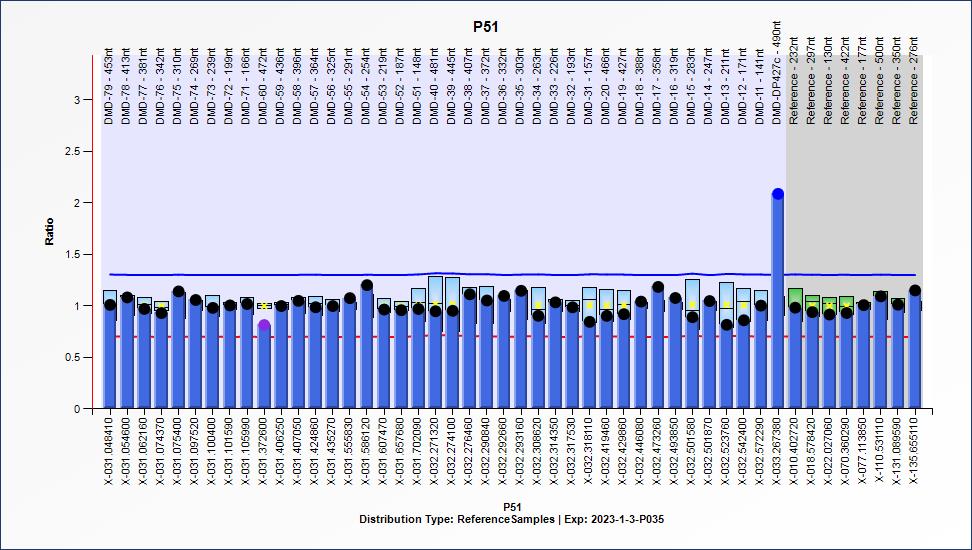


**I2：**


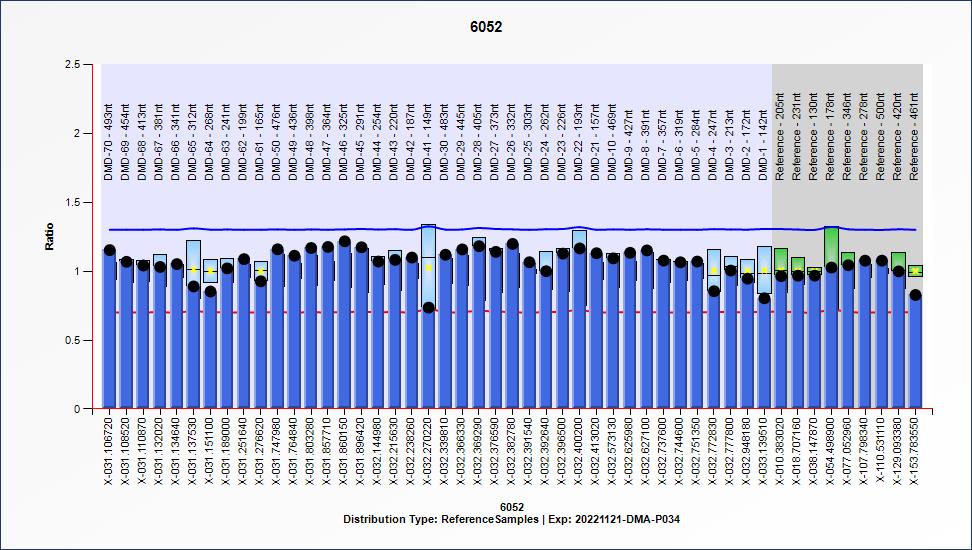

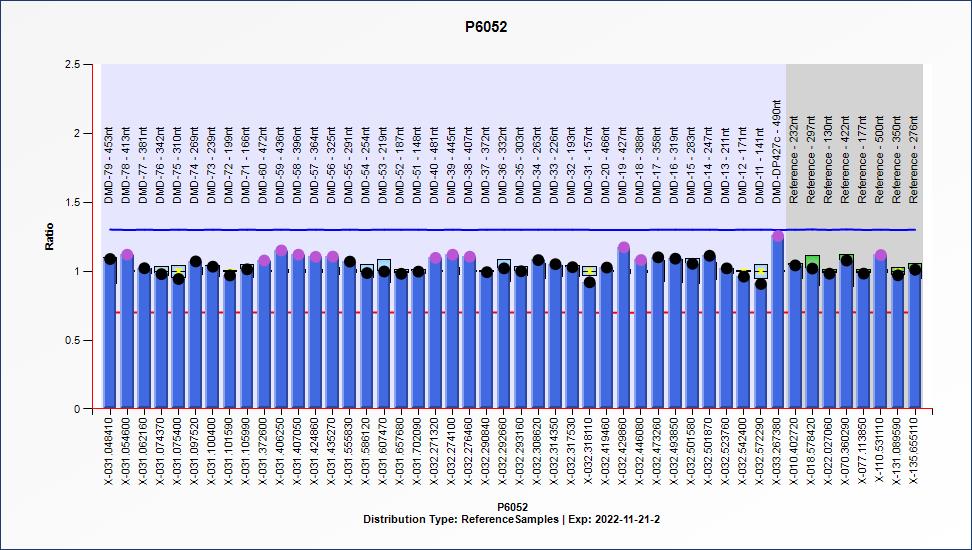


**II2：**


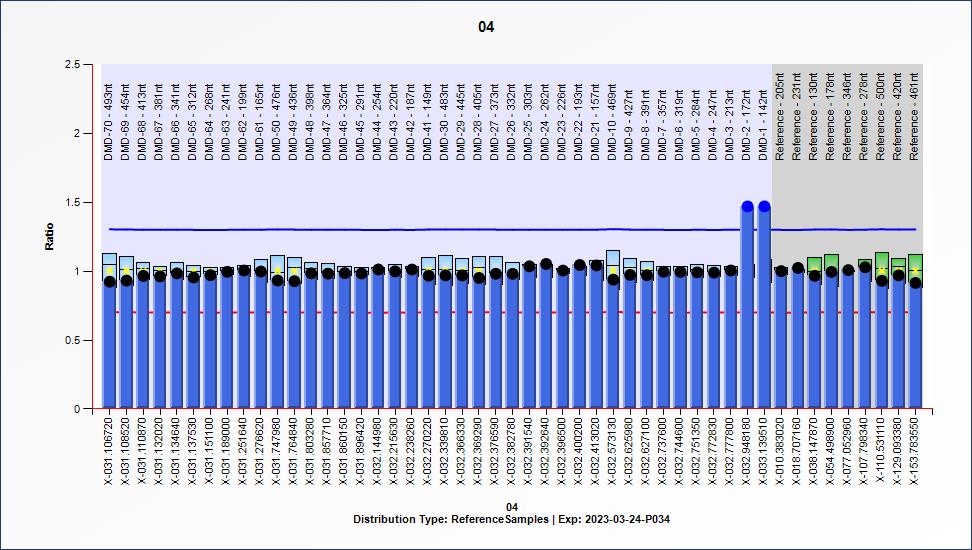

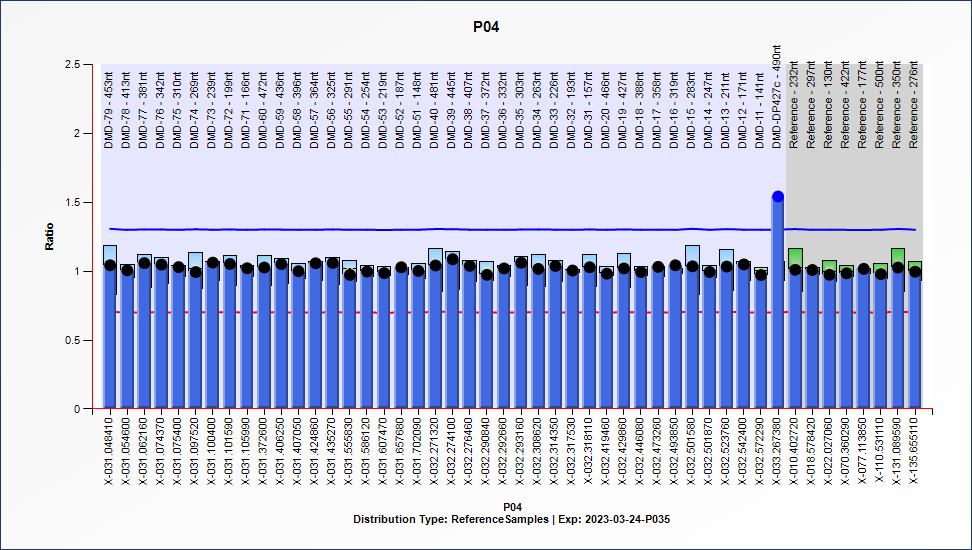


**II3:**


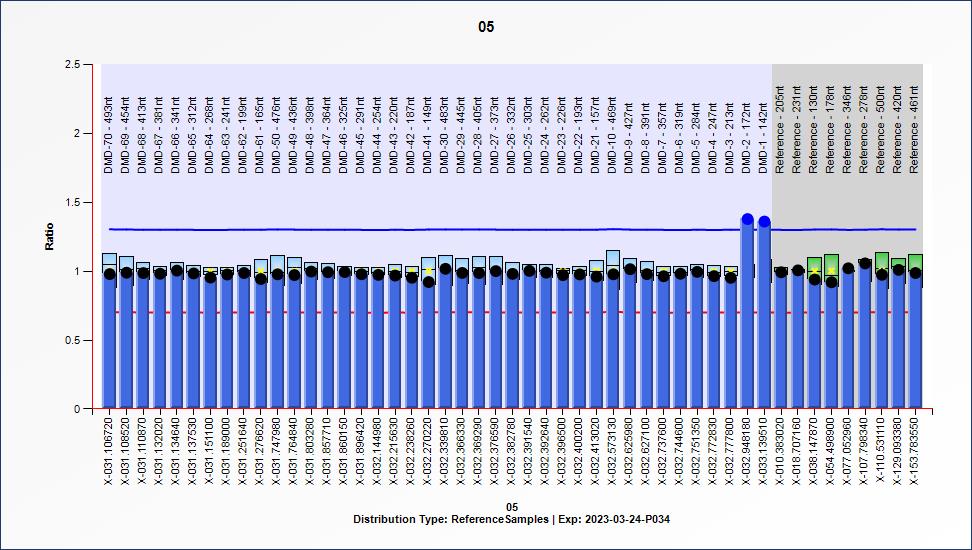

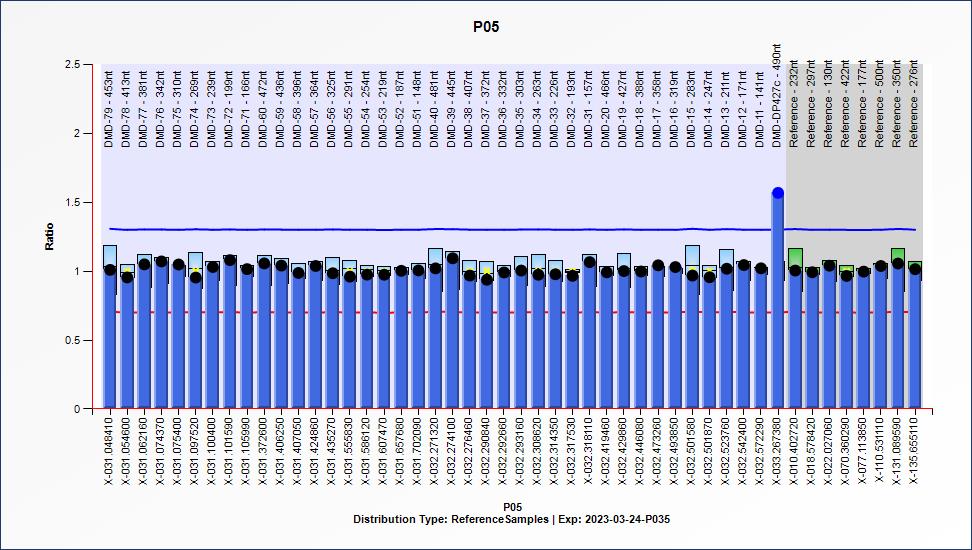


**II4：（index patient）**


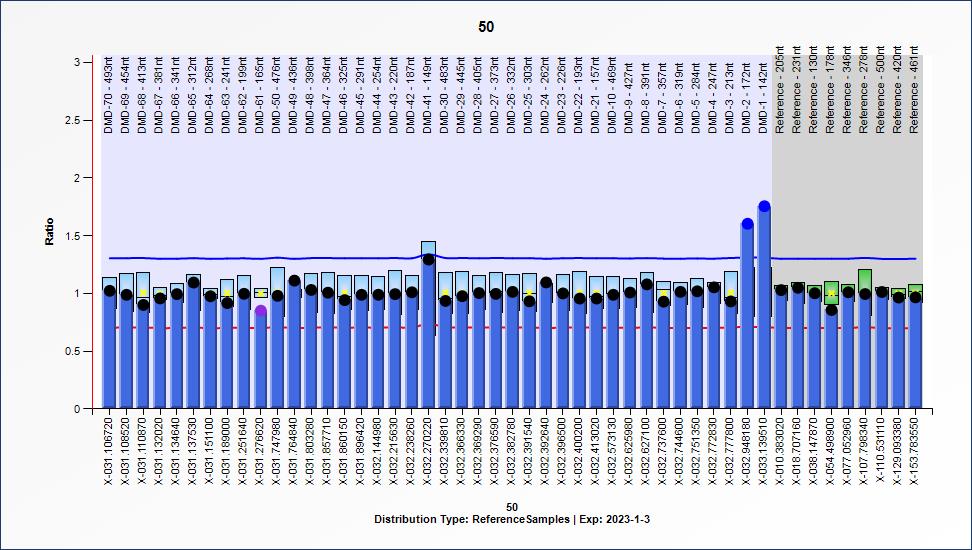

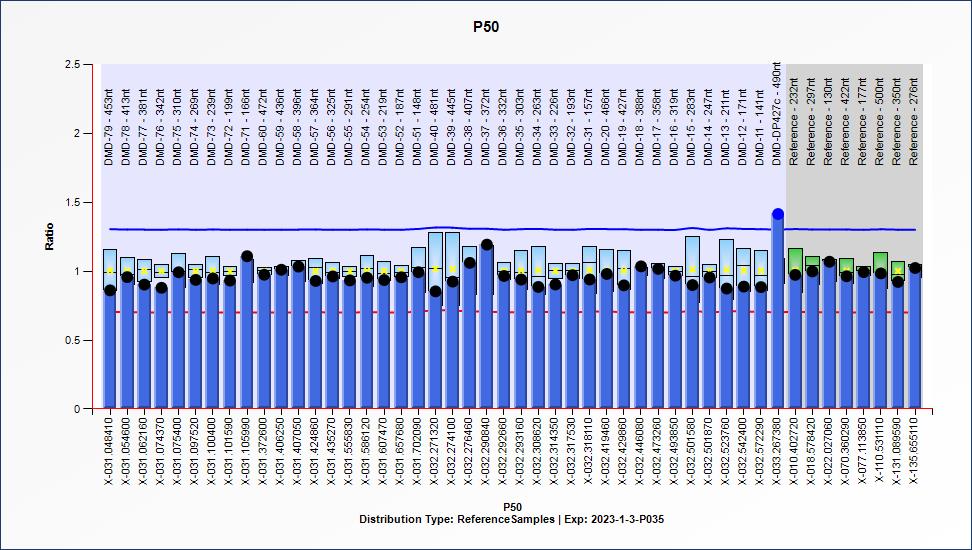


**III3:**


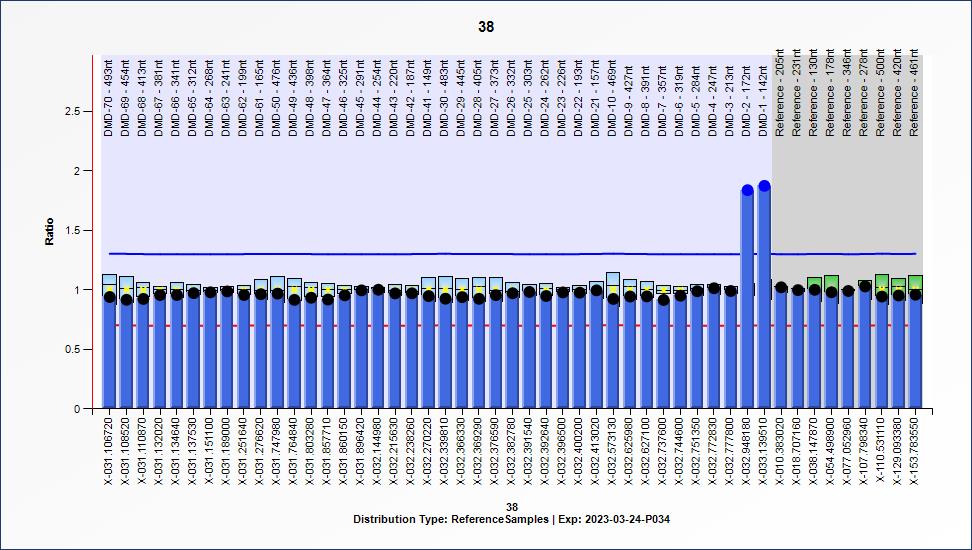

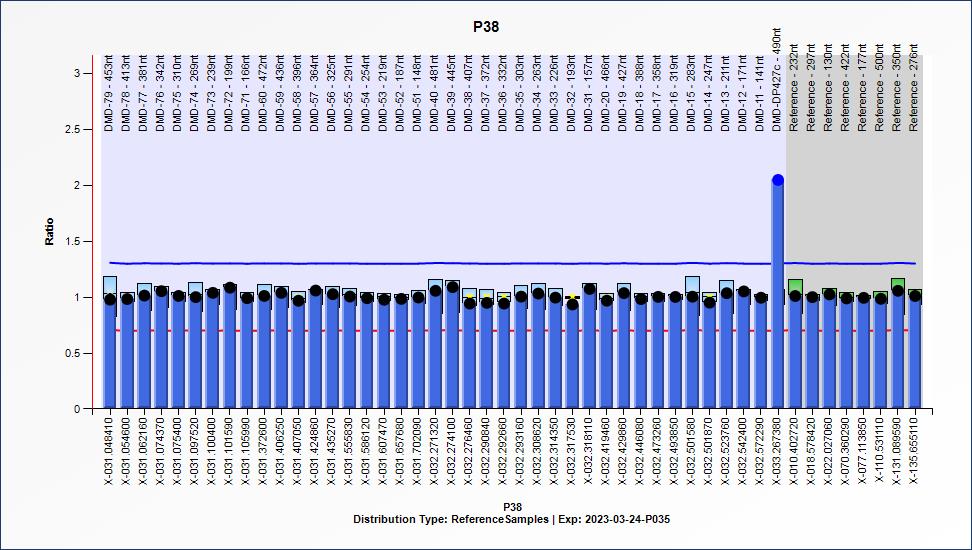


**III5:**


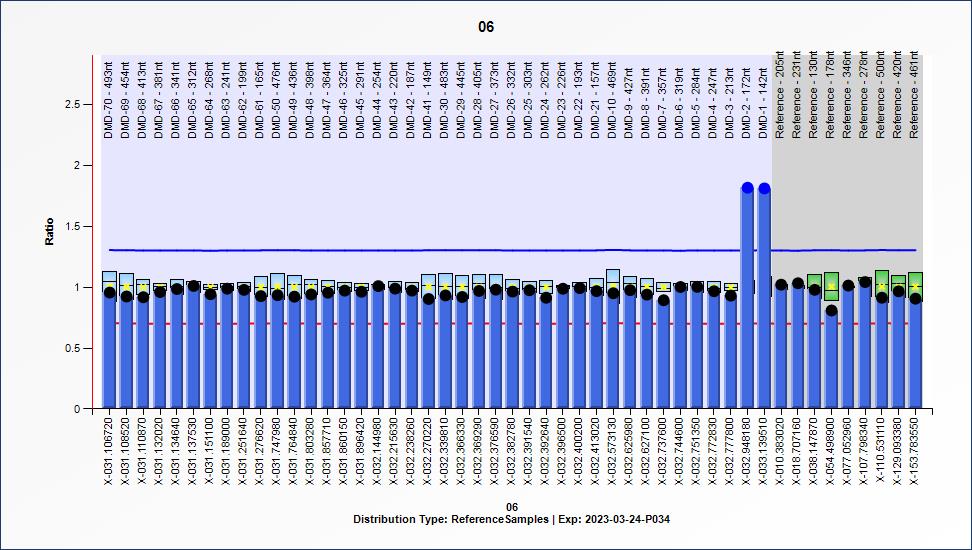

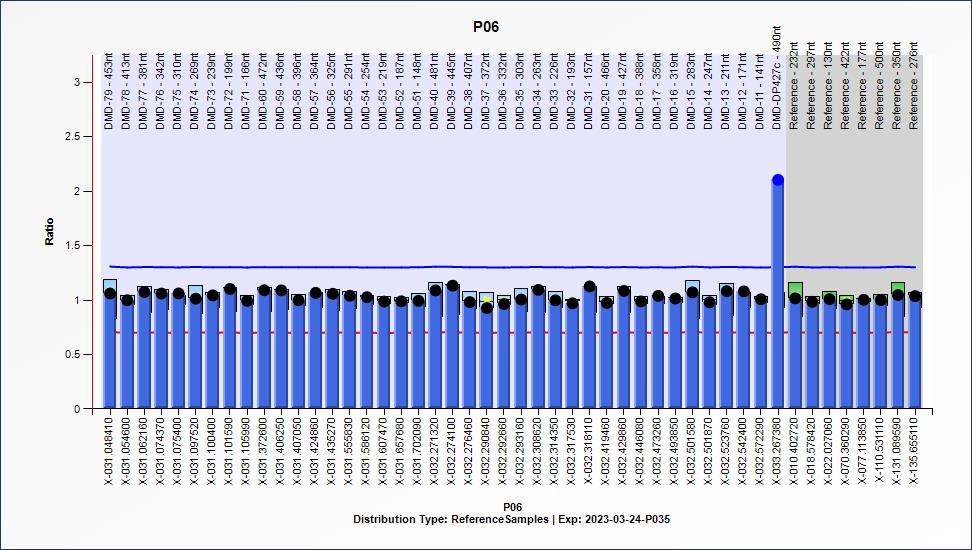


**III6：**


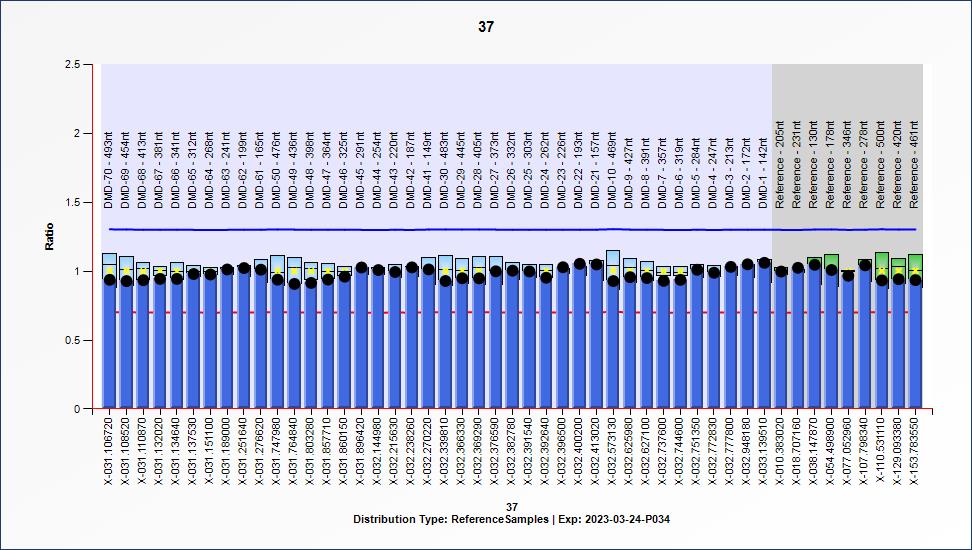

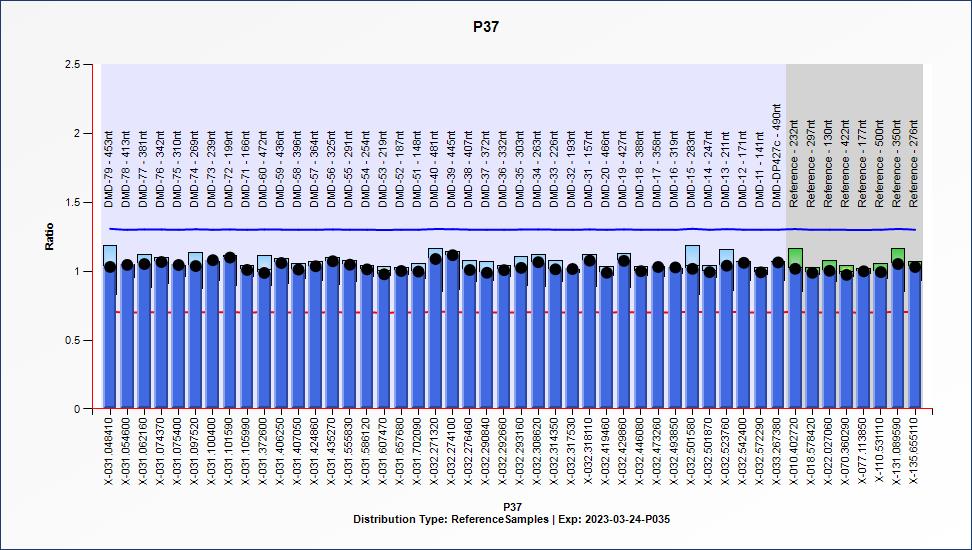


**III8**


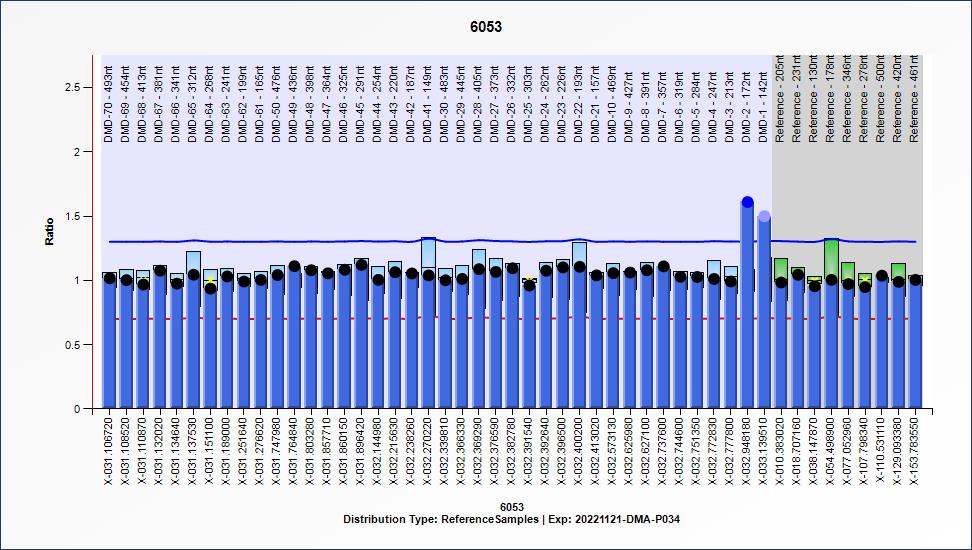

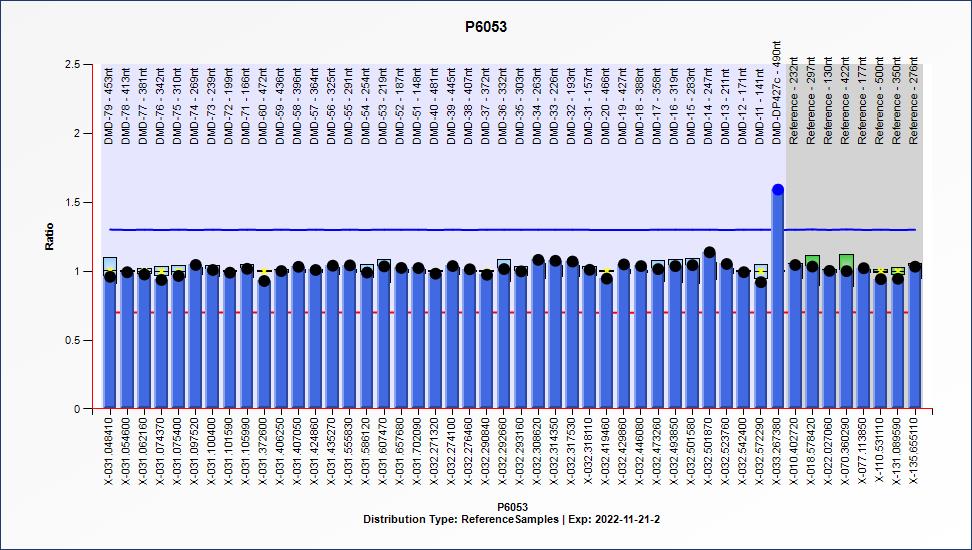

Supplement: Supplementary file 1 — Supplementary Material 1. [file 12864_2024_10224_MOESM1_ESM.docx]
