# Supplementary material for: Comprehensive analysis of genomic complexity in the 5’ end coding region of the DMD gene in patients of exons 1–2 duplications based on long-read sequencing
Source: BMC Genomics. 2024 Mar 19;25:292. doi: 10.1186/s12864-024-10224-2 (PMC10949565; doi:10.1186/s12864-024-10224-2)
Supplement: Supplementary file 2 — Supplementary Material 2. [file 12864_2024_10224_MOESM2_ESM.docx]

**Supplementary Figure 2 Copy number variants sequencing**


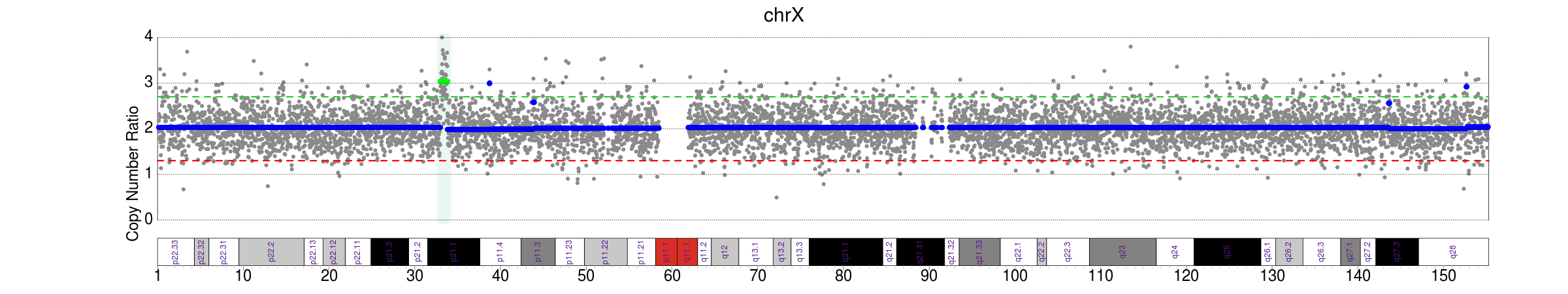


II4 in Pedigree 1: seq[GRCh37] dup(X)(p21.1) chrX:g.33020001_33840000dup


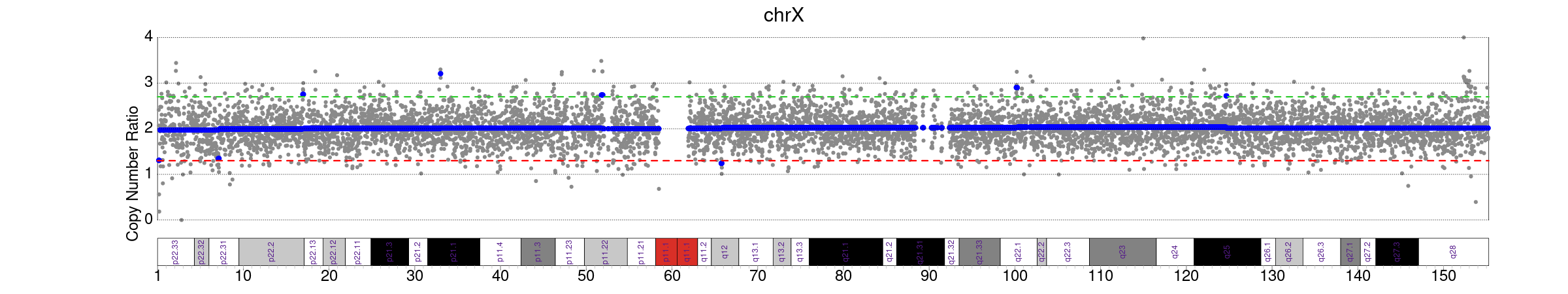


II2 in Pedigree 2: The size of the duplication segment identified by LRS(ChrX:32,999,023-33,070,000) was smaller than the resolution (100kb CNV-Seq)


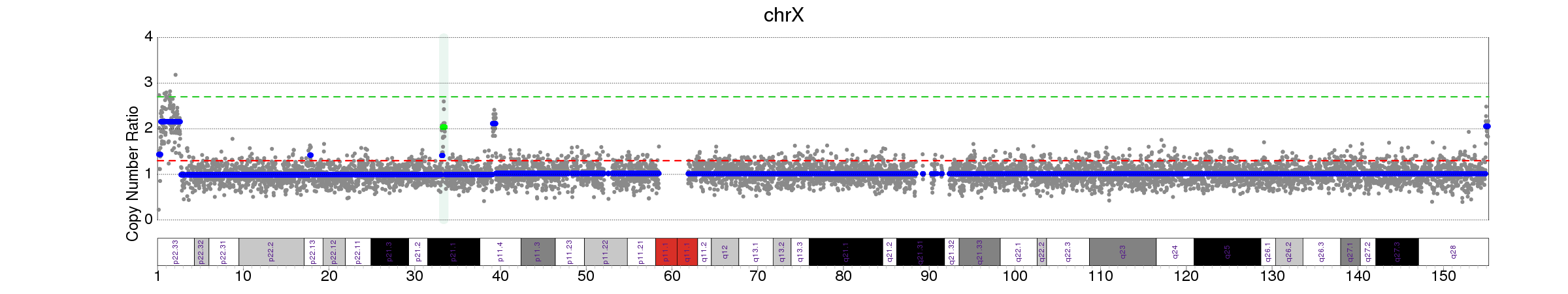


II1 in Pedigree 3: seq[GRCh37] dup(X)(p21.1) chrX:g.33280001_33540000dup, seq[GRCh37] dup(X)(p11.4) chrX:g.39080001_39480000dup.

The size of the third duplication segment identified by LRS(ChrX:33,154,320-33,202,989) was smaller than the resolution (100kb CNV-Seq).
