# Supplementary material for: Comprehensive analysis of genomic complexity in the 5’ end coding region of the DMD gene in patients of exons 1–2 duplications based on long-read sequencing
Source: BMC Genomics. 2024 Mar 19;25:292. doi: 10.1186/s12864-024-10224-2 (PMC10949565; doi:10.1186/s12864-024-10224-2)
Supplement: Supplementary file 3 — Supplementary Material 3. [file 12864_2024_10224_MOESM3_ESM.docx]

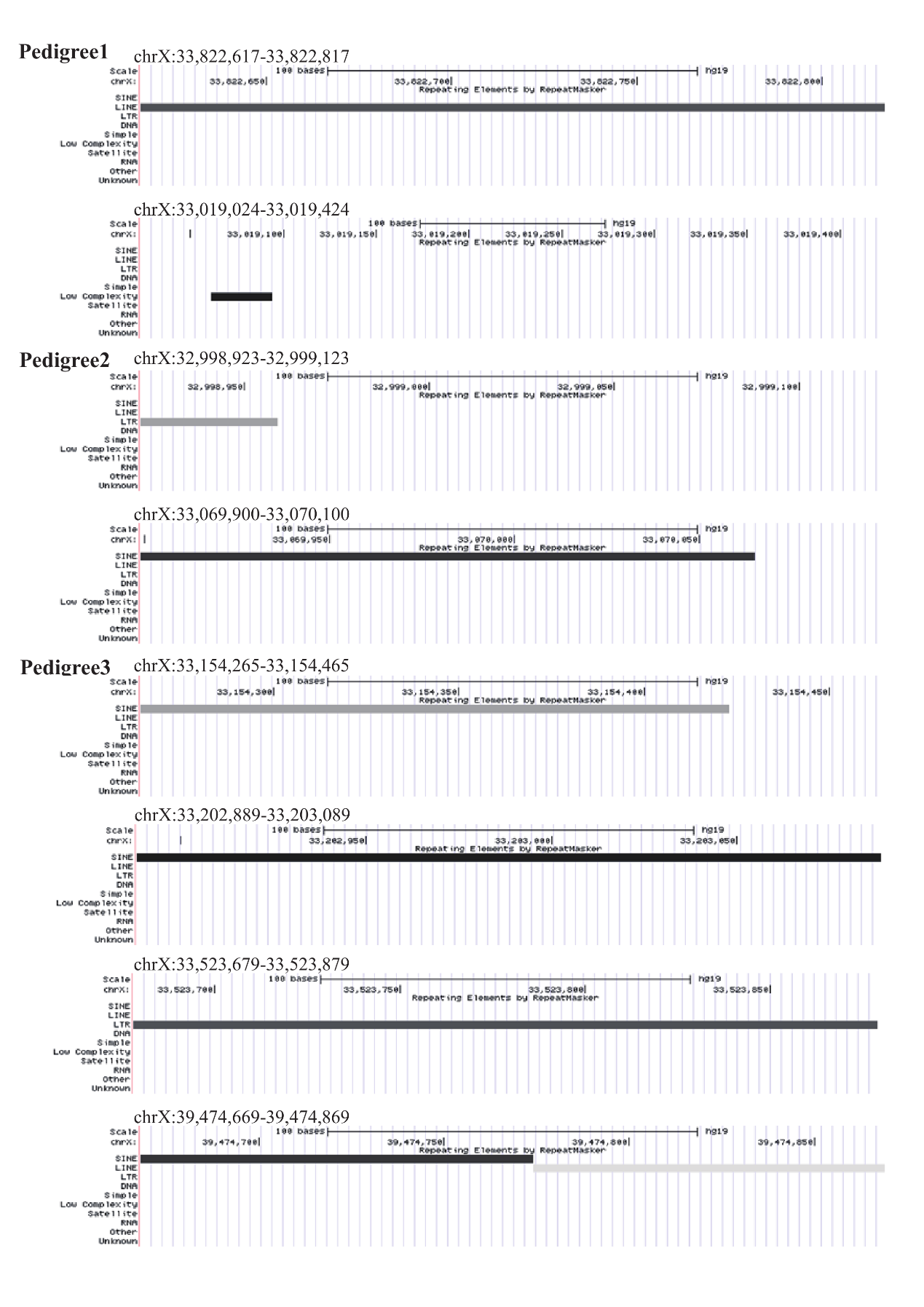
 **Supplementary Figure 3 Repeat sequences around the breakpoints**

Each inset was generated by UCSC Genome Browser (http://genome.ucsc.edu/index.html)
