# Supplementary material for: Comprehensive analysis of genomic complexity in the 5’ end coding region of the DMD gene in patients of exons 1–2 duplications based on long-read sequencing
Source: BMC Genomics. 2024 Mar 19;25:292. doi: 10.1186/s12864-024-10224-2 (PMC10949565; doi:10.1186/s12864-024-10224-2)
Supplement: Supplementary file 4 — Supplementary Material 4. [file 12864_2024_10224_MOESM4_ESM.docx]

**Supplementary Table 1 PCR primers for breakpoints validation**

| **Primer Name** | **Primer Sequence (5’→3’)** |
| --- | --- |
| Pedigree1-F | TGAGGTCCATTAGATGTTTTG |
| Pedigree1-R | AGTCACTCCTTTTGCTTGTTC |
| Pedigree2-F | GCCTCAACCGACTTTTTTAGAC |
| Pedigree2-R | GACCCTCCTTGCTTGTTGTTT |
| Pedigree3-1-F | GAGTCTCGCTCTGTCGCCCA |
| Pedigree3-1-R | TGTGGTGTTGAGCCTGTGGGT |
| Pedigree3-2-F | CCACAAAAATGGAAGTGAAGAAC |
| Pedigree3-2-R | TGCAAAAAGAAGAAGTAAAAGACA |
| Pedigree3-3-F | GGGAGGCTGAGGCACAAG |
| Pedigree3-3-R | AGTATCCCAGCATTGTTCCATTA |
