# Supplementary material for: Comprehensive analysis of genomic complexity in the 5’ end coding region of the DMD gene in patients of exons 1–2 duplications based on long-read sequencing
Source: BMC Genomics. 2024 Mar 19;25:292. doi: 10.1186/s12864-024-10224-2 (PMC10949565; doi:10.1186/s12864-024-10224-2)
Supplement: Supplementary file 5 — Supplementary Material 5. [file 12864_2024_10224_MOESM5_ESM.docx]

**Supplementary Table 2** Parameters of whole genome nanopore sequencing.

| **Sample** | **Depth(X)** | **Total bases(bp)** | **Read length N50(bp)** |
| --- | --- | --- | --- |
| Pedigree1(II4) | 24.69X | 74,063,447,893.0 | 28,875.0 |
| Pedigree2(II2) | 31.5X | 94,497,236,547.0 | 23,948.0 |
| Pedigree3(II1) | 29.16X | 87,481,108,956.00 | 44,441.0 |
